# Atrial Cardiopathy and Sympatho-Vagal Imbalance in Cryptogenic Stroke: Pathogenic Mechanisms and Effects on Electrocardiographic Markers

**DOI:** 10.3389/fneur.2018.00469

**Published:** 2018-06-19

**Authors:** Maurizio Acampa, Pietro E. Lazzerini, Giuseppe Martini

**Affiliations:** ^1^Stroke Unit, Department of Neurological and Neurosensorial Sciences, Azienda Ospedaliera Universitaria Senese, “Santa Maria alle Scotte” General Hospital, Siena, Italy; ^2^Department of Medical Sciences, Surgery and Neurosciences, University of Siena, Siena, Italy

**Keywords:** ischemic stroke, ECG, P wave, P wave dispersion, autonomic nervous system, atrial fibrosis, atrial dilation, atrial cardiopathy

## Abstract

Recently, atrial cardiopathy has emerged as possible pathogenic mechanism in cryptogenic stroke and many electrocardiographic (ECG) markers have been proposed in order to detect an altered atrial substrate at an early stage. The autonomic nervous system (ANS) plays a well-known role in determining significant and heterogeneous electrophysiological changes of atrial cardiomyocytes, that promote atrial fibrillation episodes in cardioembolic stroke. Conversely, the role of ANS in atrial cardiopathy and cryptogenic stroke is less known, as well as ANS effects on ECG markers of atrial dysfunction. In this paper, we review the evidence linking ANS dysfunction and atrial cardiopathy as a possible pathogenic factor in cryptogenic stroke.

## Introduction

About one third of ischemic strokes occurs without a well-defined etiology and is classified as cryptogenic (1). Different possible pathogenic mechanisms have been proposed (2), including the presence of subclinical atrial fibrillation (AF). Thus, the use of prolonged outpatient cardiac monitoring is currently recommended in order to detect subclinical AF (3) and to provide clues to the mechanism of stroke, leading to appropriate secondary prevention with anticoagulant drugs.

However the relationship between AF and stroke appears more complex than a simple cause-effect mechanism and it seems that AF, atrial substrate, and systemic factors interact in complex ways in the pathway leading to stroke (4). In particular, the lack of direct evidence of a causal association and a temporal relationship between AF and thromboembolic stroke in most patients suggested the hypothesis that atrial cardiopathy may underlie most strokes; thus, AF could represent only a marker of atrial dysfunction (5). Thereby, atrial cardiopathy would represent a continuum, replacing AF as a standalone disease; according to this conceptual model, different races could have different rates of rhythm disorders (AF or atrial flutter) depending on the stage of the atriopathy, with higher risk of stroke at any of these stages (6). In this view, atrial dysfunction, or cardiopathy has emerged as possible pathogenic mechanism in cryptogenic stroke and many ECG markers have been proposed in order to detect atrial substrate at an early stage (5).

We review evidence in favor of a link between atrial cardiopathy, detected with electrocardiographic (ECG) markers, and autonomic nervous system (ANS) dysfunction in order to suggest a possible pathogenic role of ANS in determining atrial substrate that favors cryptogenic stroke occurrence.

## Atrial cardiopathy and cryptogenic stroke

**Figure 1:**
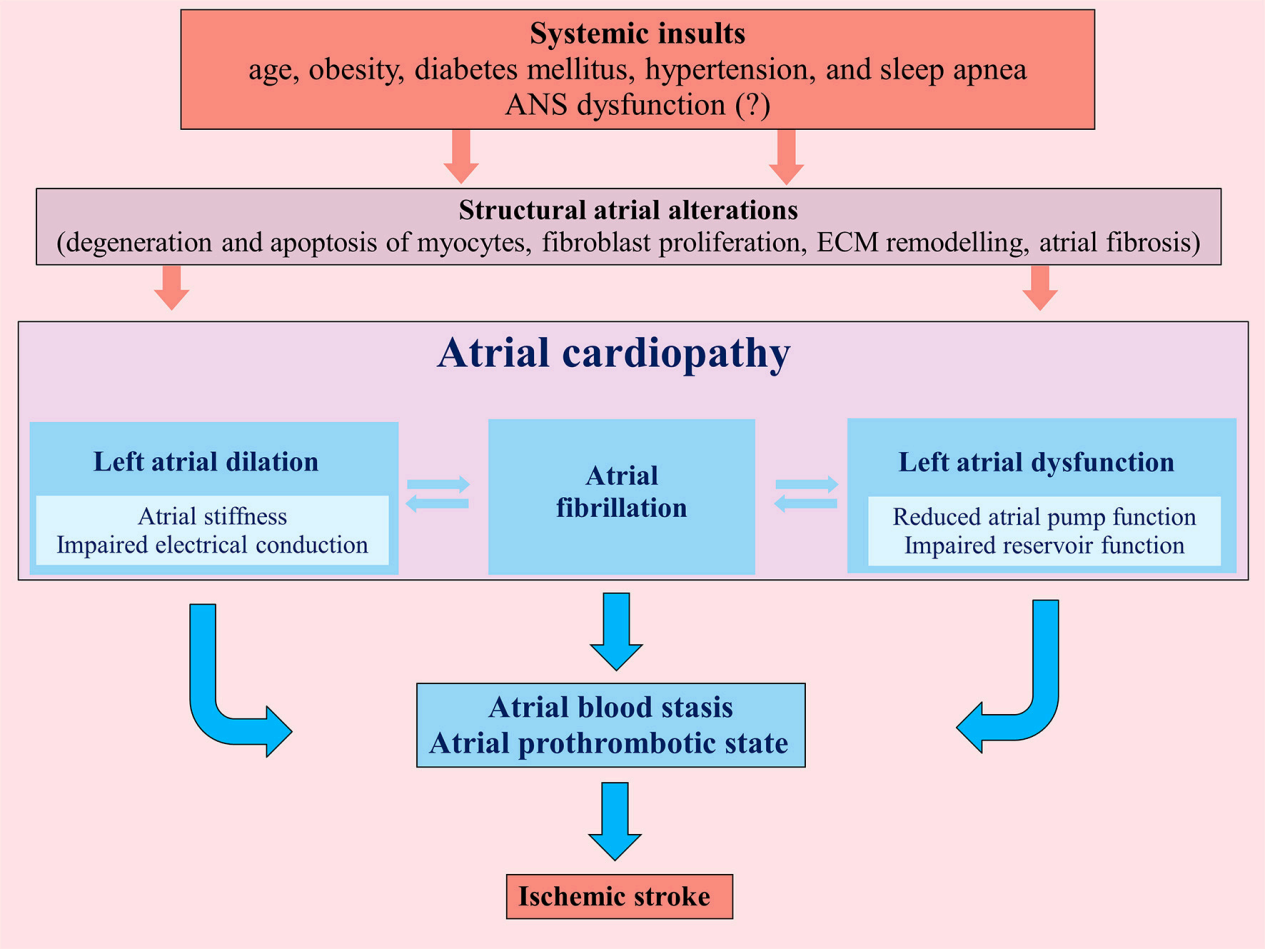
Pathogenic mechanisms determining atrial cardiopathy and favoring an atrial prothrombotic state that can lead to ischemic stroke. ECM, extracellular matrix; ANS, autonomic nervous system.

Atrial cardiopathy results from different systemic insults (age, obesity, diabetes mellitus, hypertension, and sleep apnea) that promote marked atrial histological abnormalities. These structural atrial alterations include degeneration and apoptosis of myocytes, fibroblast proliferation and differentiation into myofibroblasts with atrial fibrosis, matrix degeneration and formation of non-collagen deposits in the interstitial space (7, 8) (Figure 1).

Myocyte apoptosis promotes reparative fibrosis that replaces myocardial cells (8), whereas fibroblast proliferation induces a reactive fibrosis with an altered ratios of collagen subtypes that separate myocytes, interfering with electrical impulse propagation (8); furthermore, these structural alterations of the atrial myocardium induce the disorganization of connexins (especially Cx43) within junction channels (9).

These patho-histological changes lead to left atrial dilation and dysfunction, determining not only a substrate for AF, but also an atrial prothrombotic milieu that, by itself, represents a possible pathogenic mechanism of cardioembolic ischemic stroke, independently from AF (10) (Figure 1). Indeed atrial and left atrial appendage hypocontractility associated to atrial dilation cause blood stasis in the atrial chambers inducing a thrombotic substrate.

Left atrial dysfunction consists of reduced atrial compliance and relaxation during ventricular systole and impaired pump function during ventricular diastole; in particular atria that exhibit greater fibrotic and apoptotic burdens have impaired conduit, reservoir and contractile function (11). Reduced left atrial compliance is associated with higher clinical recurrence of AF (12) and increased left atrial stiffness has been suggested to be a predictor of cryptogenic stroke in subjects with patent foramen ovale (13). Furthermore, impaired reservoir function (assessed by left atrial reservoir strain with speckle-tracking echocardiography) is associated with cryptogenic stroke, independently of other cardiovascular risk factors (14). Finally, impaired left atrial pump function is significantly depressed in cryptogenic stroke with atrial septal aneurysm (15).

Similarly to left atrial dysfunction, left atrial enlargement is related to the degree of atrial structural pathology and the amount of atrial fibrosis; in particular, moderate-severe left atrial enlargement represents an independent marker of recurrent cardioembolic or cryptogenic stroke (16). Left atrial enlargement is also associated with high risk of AF occurrence (17), but a recent analysis of the Cardiovascular Health Study demonstrated that left atrial enlargement is associated with ischemic stroke, independently from other several confounders such as AF (16).

Primary or secondary ANS dysfunction could play a pathogenic role in atrial structural alterations leading to atrial cardiopathy. Notably, previous studies support the idea that various systemic insults (age, obesity, diabetes mellitus, hypertension, and sleep apnea) related to atrial cardiopathy could induce a secondary ANS dysfunction (18–22). In this view, ANS could represent a cause or a mediator of the pathogenic mechanisms underlying the atrial cardiopathy.

## ECG markers of atrial cardiopathy and influences of autonomic nervous system

Many ECG markers are currently used in cryptogenic stroke in order to detect the presence of a possible atrial cardiopathy (5, 23–25). They detect different aspects of atrial dysfunction and may be, at least in part, associated with ANS alterations (Table 1).

**Table 1:** Electrocardiographic markers of atrial cardiopathy and influences of autonomic nervous system.

**ECG marker of atrial cardiopathy**	**Definition**	**Effects of autonomic nervous system imbalance**
Increased P wave dispersion	The difference between the longest and the shortest P wave duration, measured in the simultaneous 12 ECG leads.	Indirect effects, (atrial fibrosis, atrial enlargement) resulting in inhomogeneous and discontinuous atrial conduction.
Increased P wave duration	The time between the onset and offset of the P wave.	Direct electrophysiological effects and indirect effects (atrial fibrosis), resulting in electrical interatrial block
Increased PR interval	The time between the onset of the P wave and the onset of the QRS complex.	Direct electrophysiological effects (modulation of cellular refractory period) and indirect effects (atrial fibrosis)
Increased P wave area	The total geometric area under the P wave in the 12-lead ECG.	Indirect effects (abnormal atrial structure and left atrial enlargement)
Increased P wave terminal force in V1	The value of the amplitude multiplied by the duration of the terminal negative portion of the P wave in lead V1 of a standard 12-lead ECG.	Indirect effects (structural atrial remodeling)
Abnormal P wave axis	The net direction of electrical forces within the atria.	Indirect effects (structural atrial remodeling) resulting in altered direction of the atrial electrical wave front propagation
Premature atrial contractions	Premature ectopic depolarizations originating in the atria.	Direct electrophysiological effects (modulation of atrial ionic channels activity) and indirect effects (structural atrial remodeling), resulting in focal ectopic firing.

### P wave dispersion

P wave dispersion results from the difference between the longest and the shortest P wave duration measured in the simultaneous 12 leads on a routine ECG (26) and has been suggested to be a marker of cardioembolism in cryptogenic stroke (27). Different P wave durations in 12-lead ECG reflect regional delays in atrial depolarization; therefore, increased PWD results from inhomogeneous and discontinuous atrial conduction based on inhomogeneous and anisotropic distribution of connections between atrial myocardial fibers (28). In this view, PWD reflects the presence of atrial substrate for AF and previous studies showed a link between high PWD values (>40 ms) and AF in cryptogenic stroke (29–31).

Increased PWD is also associated with impaired left atrial mechanical functions and enlargement in patients with cryptogenic stroke (32).

In fact, the increased collagen deposition and myocardial fibrosis of the left atrial myocardium may result in reduced LA compliance which is reflected by prolongation of the PWD on ECG (33).

The influence of ANS on PWD has been suggested by the effects of chronic traumatic spinal cord injury on ECG markers (34). Autonomic dysfunction after spinal cord injury may contribute to the development of cardiovascular disorders (cardiac arrhythmias and unstable blood pressure), especially in patients with cervical and high thoracic spinal cord injury (above T6) and it is possible that higher PWD values observed in patients with chronic spinal cord injury are linked to atrial structural alterations (34). PWD has also been suggested to be an early sign of cardiac autonomic dysfunction in subjects with neurally-mediated syncope (35).

### P wave duration

A prolonged P-wave duration (>120 ms) is considered a marker of atrial cardiopathy, reflecting the presence of an interatrial block, determined by a reduced atrial conduction related to a fibro-fatty transformation of atrial walls (36). A previous study demonstrated that in post-mortem atrial tissues from patients who died of cardiovascular causes, the extent of fibrosis, and fatty infiltration is significantly associated with prolonged P wave duration, suggesting that atrial fibrosis of Bachmann's bundle and terminal crest have a major role in prolongation of P wave duration (37). The interatrial block may be partial (when prolonged P wave duration is associated to bimodal P-wave in any lead on the standard 12-lead ECG) or advanced (when prolonged P wave duration is associated with biphasic P-wave in the inferior leads) (38).

Very long P-wave duration in the top fifth percentile is associated with doubling of risk of AF (23). A recent pooled meta-analysis showed that prolonged maximum PWD (evaluated as a categorical variable) increases the risk of ischemic stroke (24). In particular, a previous study demonstrated that in patients with advanced interatrial block there is 1.7-fold increase in the risk of ischemic stroke (39) and this association resulted not attenuated by incident AF events: this evidence suggests that left atrial disease evaluated by means of P wave duration, is possibly an independent risk factor for ischemic stroke.

The acute effects of autonomic stimulation and blockade on P wave duration are well-known electrophysiological effects (40) and are outside the scope of our review article. However, the observation that in non-elite men athletes lifetime training hours are associated with the prolonged signal-averaged P-wave duration and an increased left atrial volume suggested that a prolongation of the P wave duration reflects an altered atrial substrate, determined by the exercise-induced atrial fibrosis (41), possibly induced by increased vagal tone (42).

### PR interval

PR interval is the period of time that extends from the start of the P wave (atrial depolarization) until the start of the QRS complex (ventricular depolarization). The duration of PR interval is normally between 120 and 200 ms. In most cases, a prolonged PR interval (>200 ms) is determined by conduction delay in the atrioventricular (AV) node. Acute effects of ANS on PR interval are well-known, considering that autonomic innervation influences the conduction through the AV junction conduction by modulating the refractory period (43). However, atrial fibrosis may also be considered as the possible cause of PR interval prolongation; indeed, PR interval reproduces the atrial and AV conduction, thus P wave duration represents a relevant part of PR interval and atrial cardiopathy may contribute to the prolongation of the PR interval (44). In this view, prolongation of the PR interval is considered another possible marker of atrial disease (5) and previous studies showed the association between PR interval prolongation and AF in cryptogenic stroke (45, 46). In particular, each prolongation of 10 ms increases 30% the risk of AF in cryptogenic stroke (23); furthermore, PR interval of 200 ms or greater is associated with cryptogenic stroke and may be considered a marker of atrial cardiopathy even in the absence of AF (47).

### P wave area

P wave area (PWA) is the total geometric area under the P-wave in the 12- lead ECG, representing the product of the duration and amplitude of the P-wave and it is measured in microvolt × milliseconds (48).

PWA is considered a marker of abnormal atrial structure and left atrial enlargement. In the ARIC study, mean P wave area was a predictor of AF (49), in a recent pooled meta-analysis, higher maximal PWA increases of 10% the risk of incident stroke (24).

### P wave terminal force in lead V1

P wave terminal force in lead V1 (PTFV1) is defined as the value of the amplitude multiplied by the duration of the terminal negative deflection of the P wave in lead V1 of a standard 12-lead ECG.

This ECG marker is a predictor of AF; in particular, patients with PTFV1 in the upper 5th percentile have two-fold increased risk of incident AF (47). PTFV1 represents also a strong predictor of paroxysmal AF detection in acute ischemic stroke (50) and even in the absence of detectable AF, abnormal PTFV1 is significantly associated with incident stroke in hypertensive patients (51).

A recent metanalysis (24) showed that higher PTFV1 values increase of 59% the risk of ischemic stroke and the association between increased PTFV1 and embolic stroke of undetermined cause (52) suggested the hypothesis that left atrial cardiopathy could be involved in the pathogenesis of cryptogenic stroke even in the absence of recognized AF (53).

PTFV1 represents a sign of left atrial enlargement, but it is also associated with left atrial fibrosis, dilation and elevating filling pressure, reflecting also a delayed interatrial conduction (54). In particular, PRIMERI study demonstrated that both components of PTFV1 (amplitude and duration of the terminal portion of P wave) are differently associated with atrial alterations: the duration is strongly associated with atrial fibrosis, while the amplitude is significantly associated with indices of atrial mechanical function such as left atrial volume and left atrial strain.

Currently no studies showed an influence of ANS on PTFV1, but indirect evidence suggests a possible link between autonomic activity and PTFV1. Indeed, repeated exposure to mental stress may promote an adverse atrial remodeling and acute mental stress alters left atrial electrophysiology, increasing abnormal values of PTFV1 (55); the pathophysiology that links mental stress with PTFV1 is unknown, but a recent study suggested the possible role of ANS alterations (56).

Furthermore, sleep disordered breathing is associated with subclinical left atrial disease, as indicated by PTFV1 (57); even in this case, it is possible to hypothesize a role of ANS, because an impaired cardiac autonomic modulation in the sense of sympathetic overflow and weaker parasympathetic modulation are considered clinical hallmarks of sleep disorder breathing (58).

### P wave axis

The P-wave axis represents the net direction of electrical forces within the atria. Axis values between 0 and 75° are considered normal (48). P wave axis may be influenced by the anatomy, size and positioning of the atria within the thoracic cavity (48). Furthermore, mechanical and metabolic insults to the atria may induce structural remodeling and an abnormal electrical conduction, resulting in an altered direction of the atrial electrical wave front propagation. Therefore, an abnormal P wave axis represents a significant predictor of incident AF (59–62). Recently, the results of the ARIC study (63) showed also that an abnormal P wave axis is associated with ischemic stroke, predisposing to cardiac thromboembolism, regardless of AF occurrence.

### Premature atrial contractions

Atrial premature contractions (APCs) are premature supraventricular ectopic depolarizations originating in the atria and represent a risk marker for AF. Previous studies showed that frequent APCs and non-sustained runs of atrial tachyarrhythmia are associated with AF occurrence in cryptogenic stroke (64, 65).

In particular, APCs detected on a routine 12-lead ECG are associated with an increased risk of AF development, independently from race or sex (66) and with an increased risk for non-lacunar ischemic strokes, especially in women (67). Autonomic nervous system imbalance represents a well-known factor that is able to induce significant and heterogeneous changes of atrial electrophysiology; in particular adrenergic activation can lead to focal ectopic firing, modulating cardiac ionic channels activity and promoting atrial structural remodeling (68).

## Pathogenic role of ANS in developing atrial cardiopathy

**Figure 2:**
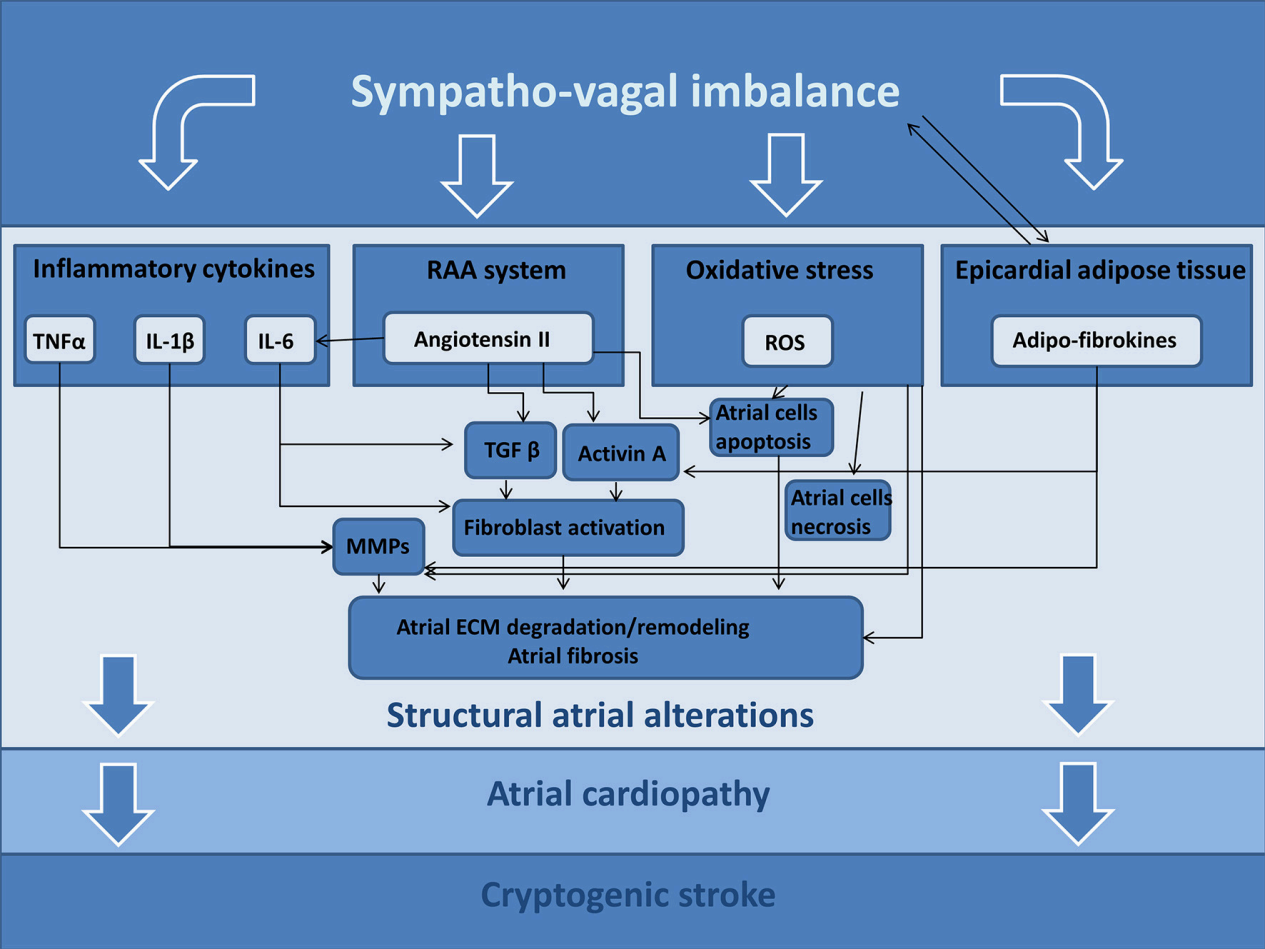
Possible ANS-related mechanisms promoting atrial cardiopathy and cryptogenic stroke. TNFα, tumor necrosis factor alpha; IL-1β, Interleukin 1 Beta; IL-6, Interleukin 6; RAA, renin-angiotensin-aldosterone; ROS, reactive oxygen species; MMPs, matrix metalloproteinases; ECM, extracellular matrix.

It is well known the role of ANS in inducing atrial tachyarrhythmias; in fact, the ANS may determine significant and heterogeneous electrophysiological changes of atrial cardiomyocytes, causing atrial fibrillation episodes (68), but these effects are out of scope for our review article. Furthermore, changes of ANS activity may trigger different signaling pathways that are able to determine an atrial derangement, promoting structural alterations. In this section, we hypothesize the possible pathogenic role of ANS in developing these structural alterations (Figure 2).

### ANS and proinflammatory cytokines

Autonomic nervous system has been suggested to affect inflammatory responses, balancing inflammatory and anti-inflammatory mediators (69), according to the so-called “inflammatory reflex” (70). In reality, the influence and the role of the ANS in inflammation are complicated by the fact that sympathetic nervous system may elicit pro-inflammatory, as well as anti-inflammatory responses according to different environments (60). A further complicating matter is the effect of local inflammation that, by itself, promotes restructuring of neuronal innervation and shifting in adrenergic receptor subtype expression (71).

Previous studies showed that ANS imbalance may induce the expression of pro-inflammatory cytokine expression in the atrial myocardial cells: in rats, a 7-day stimulation with isoproterenol leads to myocardial gene expression and protein production of tumor necrosis factor-α (TNF α), interleukin-1β (IL-1β), interleukin-6 (IL-6) (72), while beta-adrenergic blockade reduces myocardial expression of TNF-α and IL-1β, cytokines that promote impaired contractile function and chamber enlargement (73). Conversely, vagal activity exerts antiinflammatory effects on the heart, decreasing TNF-α and IL-1β (74, 75).

These cytokines are especially important in extracellular matrix (ECM) degradation; indeed, IL-1β increases the release of matrix metalloproteinases (MMP 2, 3, and 9) while TNF-α augments IL-1β-stimulated release of MMP-9 (76), that promote the net degradation of damaged ECM.

Furthermore, TNF-α induces apoptosis in cardiomyocytes (77) and apoptosis, in turn, is thought to cause atrial fibrosis by inducing a reparative fibrosis process that replaces the degenerating myocardial cells (78).

Both, TNF-α and IL-1β enhance also fibroblast migration (76), while IL-6 facilitates the conversion of cardiac fibroblasts to myofibroblasts (79), inducing the production of collagen and promoting myocardial fibrosis.

The pro-fibrotic effects of IL-6 are mediated in part by the transforming growth factor β1 (TGF-β1)/Smad3 pathway. TGF-β1 is a member of the transforming growth factor superfamily of cytokines that is able to modulate inflammatory process in atrial cells, mediating cell proliferation and differentiation; it is a potent stimulator of collagen-producing cardiac fibroblasts, inducing the production of growth factors (in particular, connective tissue growth factor), angiogenic factors (such as Platelet-Derived Growth Factor), extracellular matrix proteins, and proteases (80) and finally increasing atrial interstitial fibrosis.

Targeted gene-based reduction of TGF-β1 signaling in the posterior left atrium decreased the extent of replacement fibrosis, improving atrial dysfunction (81).

In addition to structural atrial alterations, proinflammatory cytokines (especially IL-6) may drive a prothrombotic state, leading to the increased risk of atrial thrombogenesis and, subsequently, potentially fatal thromboembolism (82, 83): proposed mechanisms linking inflammation to thrombosis include endothelial activation and/or damage, production of tissue factor from monocytes, increased platelet activation, and increased expression of fibrinogen (84).

### ANS and epicardial adipose tissue

Epicardial adipose tissue (EAT) has an important endocrine and inflammatory function, and its close relationship to the atrial myocardium suggests a role in the pathogenesis of metabolic-related cardiac diseases (85). EAT is a local source of various hormones, cytokines, and vasoactive substances affecting the myocardium (86); in particular it represents the source of several proinflammatory mediators, including IL-1β, IL-6, TNF α, monocyte chemoattractant protein-1 and adipo-fibrokines (87).

The topographic distribution of the EAT surrounding the left atrium and the lack of fascia between EAT and the myocardium enable molecules secreted by the EAT to diffuse into the myocardium, in a paracrin-like manner (88, 89). The ANS inputs to the heart converge at several locations and are typically embedded in the epicardial fat pads, forming ganglionated plexi (GP) that contain autonomic ganglia and nerves (90); thus, EAT contains both adrenergic and cholinergic nerves which interact with the extrinsic sympathetic and parasympathetic nervous system. Significant interplay occurs between the epicardial fat and the ANS; in particular, a sympathovagal imbalance is related to EAT activity and thickness (91).

The excess of EAT has a significant impact on atrial remodeling and cardiac function (92). Secretome from human EAT contains high levels of MMPs that contribute to extracellular matrix remodeling of the myocardium (76). In addition, the expression of adiponectin, a protective adipokine, is significantly lower in the EAT of patients with coronary artery disease, with evidence of inflammatory cells in the atrial tissue and local inflammatory response that may contribute to developing atrial cardiopathy. Furthermore, a recent study found that EAT secretes high levels of Activin A, an adipo-fibrokine that is able to induce atrial fibrosis: activin A may represent a possible mediator of atrial profibrotic effect and it can be neutralized by anti-Activin A antibodies (93).

### ANS and oxidative stress

Oxidative stress occurs when the formation of reactive oxygen species (ROS) and reactive nitrogen species exceeds the body's ability to metabolize them. These reactive molecules are able to determine cardiac damage by means of different molecular pathways (94): direct oxidative damage of myofibrillar proteins (95), increased inflammation by NF-κB and c-jun activation (96), TGF-β, MAPK and ERK1/ERK2 activation (96), increased activity of MMPs in cardiac fibroblasts (97), regulation of cardiomyocyte apoptosis (98). Therefore, oxidative stress represents a pathogenic mechanism promoting atrial fibrosis and structural cardiac remodeling (99).

The role of ANS in regulating oxidative stress is supported by previous evidence (100–102), showing that the increase in adrenergic drive may result in catecholamine excitotoxicity, increased oxidative stress and free radical myocardium injury. Furthermore, elevated sympathetic activity related to mental stress can lead to increased oxidative and nitrosative damage of myocardiocytes in rats (101) and sympathetic imbalance may also cause a decrease in functional respirasomes, leading to mitochondrial dysfunction and contributing to maintain and amplify the oxidative stress (102).

### ANS and renin-angiotensin-aldosterone system

The renin-angiotensin-aldosterone (RAA) system exerts a well-known role in atrial fibrosis (103). In particular Angiotensin II is a well characterized profibrotic molecule and may cause atrial fibrosis and atrial dilation by means of different mechanisms (104, 105). Angiotensin II plays an important role in the pathogenesis of atrial fibrosis via the following different mechanisms: gene expressions of profibrotic cytokines (106), including TGF1-β (107), activation of cardiac fibroblast function, upregulation of ECM protein synthesis (108), induction of atrial myocardial apoptosis (109) and activation of activin A/ALK4 activin receptor-like kinase 4 /smad2/3 pathway that plays an important role in the pathogenesis of atrial fibrosis (110).

Conversely, angiotensin converting enzyme inhibitors, angiotensin receptor blockers and aldosterone antagonists attenuate atrial fibrosis determining beneficial improvement in atrial structural alterations (105).

The ANS may modulate this system: previous studies concerning renal sympathetic denervation (RSDN) showed that interruption of afferent and efferent sympathetic signaling between the kidney and central sympathetic nervous system may reduce atrial fibrosis and atrial sympathetic nerve sprouting and these effects seem, at least in part, mediated by the suppression of renin-angiotensin-aldosteron system (111, 112).

Furthermore RSDN exerts antioxidant effects and a decrease in the local activity of the sympathetic nervous system and Angiotensin II activity (113), attenuating myocardial fibrosis and left atrial enlargement (114) and improving inflammation, apoptosis, and gap junction expression (115).

## Conclusions

In last years, atrial cardiopathy has emerged as possible pathogenic mechanism in cryptogenic stroke and many ECG markers are currently used in these patients in order to detect at an early stage the presence of a possible atrial involvement. Dissecting the role of ANS in developing atrial structural alterations leading to atrial cardiopathy may lead in the future to the development of novel therapeutic strategies to prevent atrial dysfunction and ischemic stroke.

## Author contributions

All the authors have directly contributed to drafting of the manuscript or revising it critically for important intellectual content and final approval of the manuscript submitted.

### Conflict of interest statement

The authors declare that the research was conducted in the absence of any commercial or financial relationships that could be construed as a potential conflict of interest.
